# Facing HIV infection and unintended pregnancy: Rakai, Uganda, 2001–2013

**DOI:** 10.1186/s12905-018-0535-y

**Published:** 2018-02-27

**Authors:** Stephanie Ann Grilo, Xiaoyu Song, Tom Lutalo, Margo Mullinax, Sanyukta Mathur, John Santelli

**Affiliations:** 1 Department of Sociomedical Sciences, Columbia University Mailman School of Public Health, 722 W 168th Street, New York, NY 10032 USA; 2 Heilbrunn Department of Population and Family Health, Columbia University Mailman School of Public Health, New York, NY USA; 3 Rakai Health Sciences Program, Kalisizo, Uganda; 4 American Jewish World Service, New York, NY USA; 5 Population Council, Washington, DC, USA; 6 Icahn School of Medicine at Mount Sinai andThe Tisch Cancer Institute, Icahn School of Medicine at Mount Sinail, New York, USA

**Keywords:** HIV/AIDS, Unintended pregnancy

## Abstract

**Background:**

Unintended pregnancy is a persistent and global issue with consequences for the health and well-being of mothers and babies. The aim of this paper is to examine unintended pregnancy over time in the context of substantial human immunodeficiency virus (HIV) prevalence and increasing access to anti-retro viral therapy (ART).

**Method:**

Data are from the Rakai Community Cohort Study (RCCS) – a cohort of communities with 10,000–12,000 adults, ages 15–49, in Rakai District, Uganda. We examined prevalence of current pregnancies over time, intended pregnancy, and unintended pregnancies (unwanted, mistimed, ambivalent). We then examined risk factors for the different categories of unintended pregnancy among women who were currently pregnant. The full sample included 32,205 observations over 13 years.

**Results:**

The prevalence of mistimed pregnancy and unwanted pregnancy both decreased significantly over time (*p* < .001). The prevalence of current pregnancies and intended pregnancy showed no significant changes over the thirteen year period. The same overall pattern was found when only examining HIV positive women in the sample; however, the trends were not significant. Out of the 2820 current pregnancies reported, 54.4% were intended, 29.8% were mistimed, 13.2% were unwanted, and 2.5% were ambivalent. After controlling for other predictors, HIV status had no independent effect on mistimed pregnancy but had a significant effect on unwanted pregnancy (RRR = 2.44, 95% CI = 1.65–3.61, *p* < .001] and ambivalent pregnancy [RRR = 2.07; CI: 1.03 to 4.18, *p* = 0.041]. In 2004, after the introduction of ART, there was a decreased risk in unintended pregnancy [RR = 0.75; CI: 0.66 to 0.84, *p* < .001]. Women with a secondary education or higher also had a decreased risk in unintended pregnancy [RR = 0.70; CI: 0.70 to 0.92, *p* = 0.002].

**Discussion:**

HIV was an important predictor of unwanted pregnancy. Unintended pregnancy decreased in the sample over time which may be due to an increase in ART availability and rising levels of education.

## Background

Unintended pregnancy is a persistent and global issue with considerable consequences for the health and well-being of mothers, babies, and families [1]. The Committee on Unintended Pregnancy of the Institute of Medicine declared that “the consequences of unintended pregnancy are serious, imposing appreciable burdens on children, women, men and families” [2]. In 2012, 213 million pregnancies occurred worldwide, with an estimated 85 million classified as unintended [3]. Unintended pregnancies included mistimed births (wanted if occurred later), unwanted births (not wanted now or later), and induced abortions. Women often express divided, conflicted, or ambivalent feelings about specific pregnancies.

Antecedent factors including demographic (e.g. age, parity), relationship (e.g. marital status, partner preferences) and social factors (e.g. poverty and family support) are associated with unintended pregnancy. There are many potential consequences of unintended pregnancy such as delay in initiation of prenatal care and infant health care, increased risk of child mortality, and increased risk of maternal depression and anxiety [5]. Due to the illegality of abortion in Uganda, unintended pregnancy may also lead to illegal and unsafe abortion which may result in infection and maternal morbidity and mortality [6].

High rates of unintended pregnancy and substantial HIV prevalence co-exist in the Ugandan population [4, 8]. Contextually, there are over 22 million people infected with HIV in Sub-Saharan Africa who are at reproductive age [4]; therefore, there is a growing body of literature focused on the relationship between HIV status and pregnancy desire given the increasing availability of antiretroviral therapy (ART) and prevention of mother-to-child transmission (PMTCT) [9–13].

Some studies have demonstrated an impact of HIV status on pregnancy intentions while others have not, however a major limitation of these studies are cross-sectional research designs. For example, a previous study conducted in Rakai, Uganda showed that the majority of women who were HIV positive did not want more children, yet were at high risk for unintended pregnancy due to their reported sexual behaviors [9]. HIV status may impact women’s pregnancy intentions as they may be afraid of mother-to-child transmission or becoming sicker. Recent research in Uganda also demonstrates that women on ART with higher optimism scores have increased fertility desires and pregnancy intentions [10–12]. A qualitative study in rural Uganda found that HIV positive women reported that most of their pregnancies came as a surprise, and many believed they were infertile because of HIV [13].

The circumstance of HIV in Uganda has shifted dramatically over the last decade; therefore, it is important to examine the effects of these changes on unintended pregnancy. Using longitudinal data, this paper will explore the trends in classification of pregnancies as intended or unintended with data from 2001 to 2013. During this period, HIV treatment and prevention have become available including PMTCT in the early 2000’s, antiretroviral treatment (ART) starting in 2004, and male circumcision (MC) in 2008. Since the implementation of these HIV programs, fertility desire (wanting a pregnancy in the next 12 months) increased among both HIV-positive and HIV-negative women in Rakai [14]. Relatively stable HIV incidence coupled with improved treatment options means that today many people of reproductive age will live with HIV for many years. The acknowledgement and understanding that there are multiple dimensions of ‘unintendedness’ is necessary in order to understand the impact of unintended pregnancy on health [5]. Even though the complexity of unintended pregnancy is well known [7], most research looking at the relationship between HIV and unintended pregnancy is only able to examine intended versus unintended.

This study has two main aims. The first is to examine trends in unintended pregnancy over time – in the face of an evolving epidemic of HIV in Uganda. Due to the unique longitudinal data RCCS collects, we are able to look at the change of HIV prevention and treatment programs and how treatment for HIV may affect women’s report of pregnancy intentions.

The second major aim of this study is to compare HIV as a risk factor for unwanted, mistimed, and ambivalent unintended pregnancies. We hypothesize that there will be differences in predictors of of these categories of unintended pregnancy. Thus, we explored differences between intended and unintended pregnancies and among the categories of unintended pregnancy– mistimed, unwanted, or ambivalent.

## Methods

### Study design

The data for this paper comes from women in the Rakai Community Cohort Study (RCCS) [15]. We used longitudinal data from RCCS rounds 8–15, which were conducted between 2001 and 2013, from 28 communities in Rakai District, Uganda that were followed continuously between 2001 and 2013. Each survey round lasts between a year and year-and-a-half. Each survey round of data collection includes a household census, individual surveys, and biomarker data.

The household census measures household characteristics and enumerates household members based on mortality, fertility, and migration There are baseline surveys for first time participants and follow-up surveys for previously enrolled participants. Households are recruited based on a census of each community. If members of the household pass the study criteria (e.g. age, consent), they are recruited for participation. If a participant moves out of the household into one of the other RCCS communities, he/she is tracked and invited to participate in the next survey round. If the participant moves out of the region for the survey cycle, a record is made and his/her information is entered as missing.

The individual survey collects socio-demographic, biological, and behavioral data from participants. Interviewers receive extensive training and are matched to the sex of the interviewee. Interviewers administer the survey in a confidential face-to-face interview. During the face-to-face interview, the participant provides biological samples to test for HIV and sexually transmitted infections (STIs). Ethical approvals for RCCS for data collection and for this analysis were obtained from the Institutional review boards (IRBs) at Columbia University Medical Center and the Uganda Viral Research Institute.

Selection criteria included being female, being currently pregnant, and answering the questions on pregnancy intention, which is only asked in the follow-up survey. These criteria yielded 2820 pregnancies with 2218 unique women. We limited the sample to current pregnancies to have a clear order of predictor and outcome variables, including current HIV status.

To explore trends over time, we calculated pregnancy prevalence and prevalence of women reporting four ‘types’ of pregnancy: intended, mistimed, unwanted, or ambivalent. Pregnancy prevalence is reported as the percentage of women currently pregnant at each round. The prevalence of each ‘type’ of pregnancy is the number of women reporting each type divided by total number of women in the sample. To assess the risk factors associated with the four ‘types’ of pregnancy, we used the proportion currently pregnant women who reported intended, mistimed, unwanted or ambivalent pregnancies.

### Variables of interest

#### Outcome variable

The main outcome variable in this analysis is intended or unintended pregnancy among currently pregnant women. Women who are currently pregnant are asked, “Did you intend to have this pregnancy at this time?” If a woman reports an unintended pregnancy, she is then asked, “Was this pregnancy just mistimed, totally unwanted, or did you not care whether it happened or not.” “Did not care” was labeled “ambivalent.” We used the dichotomous measure intended vs. unintended in the first set of analyses. We then used the categorical variable of mistimed, unwanted, or ambivalent for the next set of analyses.

#### HIV serostatus

A primary predictor of interest was HIV serostatus. HIV status is collected from all participants who agree to provide a blood sample (> 90% of participants agree to provide a blood sample). Previously, HIV status was determined by two enzyme-linked immunosorbent assays confirmed by Western blot. More recently, HIV serostatus is determined in the field from a validated three assay rapid test algorithm – Determine and Stat Pak are run in parallel with Unigold as the tiebreaker [16, 17]. All new rapid test positives, and discordant/weak positive bands are assessed using two enzyme immunoassays (EIAs: Vironostika and Bio Rad). Polymerase chain reaction (Abbott RealTime HIV-1 PCR) confirmation is conducted on new seroconverters or samples with discordant EIAs.

#### Other variables

The demographic characteristics from this sample were collected through the household census and RCCS survey. In 2004, ART was introduced to the community. We included a variable of pre-2004 and post-2004 in our model to measure the potential impact of ART. Individual characteristics included: age categorized as 15–19, 20–24, 25–29, 30–34, 35–39, 40–49), number of living children categorized as 0, 1–2, 3–4, 5+, and educational attainment categorized as no school, primary education, and secondary education or higher. Socioeconomic status (SES)- categorized as low, middle, and high – was based on a series of questions about household possessions such as radios and the use of modern building materials to construct house [18]. Relationship variables included marital status categorized as never married, currently married, or previously married, and number of lifetime sexual partners categorized as 0–1, 2–3, 4 + .

### Analyses

In order to examine characteristics of the sample by unintended pregnancy outcomes, descriptive analyses were performed. Poisson regression analysis was used to test associations between pregnancy intention status (intended vs. unintended), individual and household characteristics (age, parity, SES), HIV status, and relationship factors (marital status, number of sexual partners in the lifetime). Poisson regression was chosen because the prevalence of unintended pregnancy is high in the sample and this method provides similar estimates to relative risk. Poisson regression was used in previous papers that used the same data set [19]. Multinomial logistic regression was then performed to test the associations between pregnancy intentions (mistimed, unwanted, and ambivalent) and the same set of individual characteristics used in Poisson regression. Multinomial logistic regression was chosen because the outcome has multiple categories. Analyses were conducted using STATA version 14.

The data were longitudinal with subjects repeatedly measured; therefore, longitudinal data analyses techniques were used to account for correlation between subject responses. Generalized estimating equations (GEE) with independent correlation structure were used to obtain robust standard errors and *p*-values.

## Results

### Pregnancy prevalence trends

**Fig. 1:**
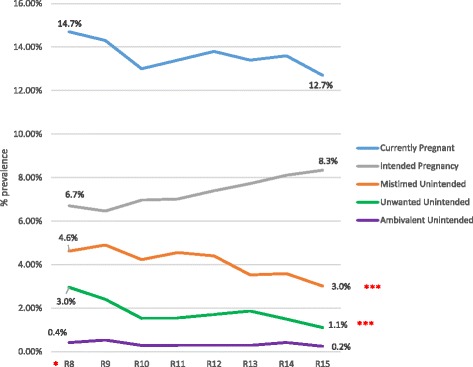
Prevalence of current pregnancy over time by intention status. *R8-R15 = Round 8 to Round 15. ***p<.001

The prevalence of pregnancy in our sample was 14.7% in round 8 and 12.7% in round 15; these numbers are not significantly different (*p* = 0.128). The prevalence of intended pregnancy (calculated by multiplying the percent intended among pregnant women during that round times the overall prevalence of pregnancy during that round) was 5.48% in round 8 and 6.67% in round 15 (*p* = 0.392). The prevalence of all three types of unintended pregnancies decreased over time with unwanted pregnancies decreasing from 2.95% in 2001–2003 to 1.1% in 2011–2013 (*p* < .001) and mistimed pregnancies decreasing from 4.6% in 2001–2003 to 3.0% in 2011–203 (*p* < .001). The trend for ambivalent pregnancies was also downward, but not significantly. See Fig. 1 for pregnancy intentions over time.

**Fig. 2:**
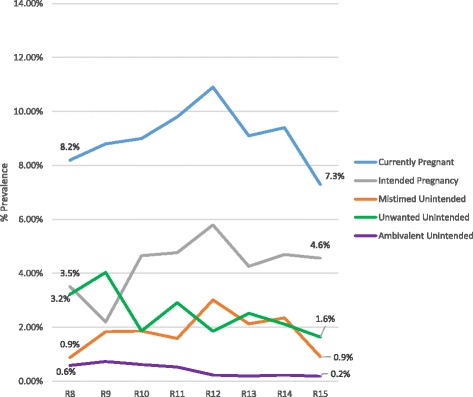
Prevalence of current pregnancy over time by intention status for HIV positive women

We also tested the change over time in unintended pregnancy for HIV-positive women (shown in Fig. 2). The prevalence of pregnancies among HIV-positive women remained relatively constant over time – from 8.2% in 2001–2003 to 7.3% in 2011–2013 (*p* = 0.314). The prevalence of intended pregnancies showed an increasing trend but was not significant. The prevalence of unwanted, mistimed, and ambivalent pregnancies showed a decreasing trend but again were not significant.

### Sample characteristics

Descriptive analyses by pregnancy intention status (intended, mistimed, unwanted, and ambivalent) are reported in Table 1. Out of the 2820 reports of current pregnancies, 54.4% were intended, 29.8% were mistimed, 13.2% were unwanted, and 2.5% were ambivalent.

**Table 1:** Sample characteristics by pregnancy intention status, Rakai community cohort study, Uganda 2001–2013

Characteristics	*N*	Intended Pregnancy	Mistimed	Unwanted	Ambivalent	*P*-value
	*N* = 2820	*N* = 1535	*N* = 841	*N* = 373	*N* = 71	
		54.4%	29.8%	13.2%	2.5%	
Sample characteristics						
Pre or Post ART	2820					*p* < .001
Pre 2004	809	45.7	33.6	17.6	3.1	
Post 2004	2011	57.9	28.3	11.5	2.3	
Individual characteristics						
Age (years)	2820					*p* < .001
15–19	240	50.8	40.0	6.3	2.9	
20–24	897	60.4	34.8	2.9	1.9	
25–29	844	57.0	29.9	10.1	3.1	
30–34	535	51.2	23.9	23.4	1.5	
35–39	235	42.6	19.6	35.3	2.6	
40–49	69	23.2	10.1	56.5	10.1	
SES	2819					*p* < .001
Low	664	48.0	33.4	16.0	2.6	
Middle	914	52.1	31.4	13.7	2.8	
High	1241	59.6	26.8	11.4	2.3	
Education	2820					*p* < .001
No school	128	42.2	35.2	19.5	3.1	
Primary school	1786	49.9	32.1	15.3	2.6	
Secondary or higher	906	65.0	24.5	8.3	2.2	
HIV Status	2820					*p* < .001
Positive	292	49.0	20.2	26.7	4.1	
Negative	2528	55.1	30.9	11.7	2.3	
Relationship risk variables						
Parity	2820					*p* < .001
0	219	74.4	20.6	2.7	2.3	
1 or 2	931	59.3	32.9	5.3	2.6	
3 to 4	600	50.2	33.7	13.7	2.5	
5+	1070	48.5	26.9	22.1	2.5	
Currently married	2820					*p* < .001
Never married	250	57.2	32.0	8.8	2.0	
Married	2356	54.8	30.4	12.3	2.4	
Separated or widowed	214	46.7	20.6	28.5	4.2	
Number of Sexual Partners Ever	2820					*p* < .001
0–1	729	51.9	35.3	10.7	2.2	
2 or 3	1592	56.5	29.6	11.3	2.6	
4+	499	51.7	22.7	23.1	2.6	

The traditional patterns and predictors of unintended pregnancy were found in our analyses. Women ranged in age from 15 to 49 years with a mean age of 26.7 (SD = 5.8). Not surprisingly, the age group with the highest percentage of intended pregnancy was 20–24 years. The youngest age group had a higher percentage of mistimed pregnancies; whereas, the older age groups had higher percentages of unwanted and ambivalent pregnancies. Women in households with high socioeconomic status also had higher percentages of intended pregnancy (59.6%) as compared to middle (52.1%) and low (48.0%). Women with five or more previous children were less likely to report an intended pregnancy (48.5%) than those with 3–4 children (50.2%), 1–2 children (59.3%) or 0 children (74.4%). Women who have never been married had the highest percentage of intended pregnancy (57.2%); separated or widowed women had the highest percentage of unwanted pregnancies (28.5%). Women with the most partners, 4 or more, showed the lowest percentage of intended pregnancies (51.7%) and the highest percentage of unwanted pregnancies (20.0%).

10.4% (*N* = 292) of the women in our sample were HIV positive. More currently pregnant, HIV positive women reported that their last pregnancy was intended (49.0%) compared to mistimed (20.2%), unwanted (26.7%) and ambivalent (4.1%) pregnancies. Women who were HIV positive and women who were HIV negative reported similar percentages for intended pregnancy and ambivalence about a pregnancy. HIV-positive women, compared to HIV-negative women, were less likely to report that the pregnancy was mistimed (20.2% vs. 30.9%) and more likely to report that the pregnancy was unwanted (26.7% vs. 11.7%).

### Poisson regression model:

Table 2 shows the adjusted and unadjusted Poisson models comparing intended and unintended pregnancies. The adjusted model in Table 2 is the final model with all significant predictors, using backward selection. A risk ratio above 1 indicates an increased risk for the outcome which in this case is the risk of an unintended pregnancy. A risk ratio below 1 indicates a decreased risk.

**Table 2:** Predictors of Unintended Pregnancy Using Poisson Regression, Rakai Community Cohort Study, Uganda 2001–2013

	Poisson Regression (Unadjusted)				Backwards Selection Multiple Poisson Regression (Adjusted)			
					*n* = 2819			
RISK FACTORS	Unintended v. Intended				Unintended v. Intended			
	Risk Ratio	95% Lower	95% Upper	*P*-value*	Risk Ratio	95% Lower	95% Upper	*P*-value*
Sample Characteristics								
Pre or Post ART								
pre 2004	ref	ref	ref	ref	ref	ref	ref	ref
post 2004	0.78	0.69	0.87	<.001	0.75	0.66	0.84	<.001
Individual Characteristics								
Age								
15–19	1.14	0.93	1.41	0.207	1.37	1.09	1.73	0.007
20–24	0.92	0.79	1.07	0.265	0.99	0.85	1.16	0.948
25–29	ref	ref	ref	ref	ref	ref	ref	ref
30–34	1.13	0.97	1.33	0.121	1.07	0.91	1.26	0.414
35–39	1.34	1.10	1.63	0.004	1.23	1.00	1.51	0.048
40–49	1.79	1.34	2.38	<.001	1.54	1.14	2.07	0.005
Education								
No school	1.15	0.91	1.44	0.234	1.10	0.87	1.40	0.411
Primary school	ref	ref	ref	ref	ref	ref	ref	ref
Secondary schol or more	0.70	0.61	0.79	<.001	0.80	0.70	0.92	0.002
SES								
low	ref	ref	ref	ref	ref	ref	ref	ref
middle	0.92	0.8	1.06	0.261	0.99	0.86	1.14	0.899
high	0.78	0.68	0.89	<.001	0.91	0.78	1.05	0.177
HIV Status								
Positive	1.14	0.96	1.35	0.145	1.14	0.96	1.35	0.149
Parity								
0	0.63	0.47	0.83	<.001	0.61	0.45	0.81	<.001
1 or 2	ref	ref	ref	ref	ref	ref	ref	ref
3 or 4	1.22	1.05	1.42	0.009	1.25	1.06	1.47	0.009
5+	1.26	1.11	1.44	<.001	1.29	1.11	1.51	<.001
Marriage Status								
never married	0.95	0.78	1.16	0.596				
married	ref	ref	ref	ref				
separated or widowed	1.18	0.97	1.43	0.094				
Number of Sex Partners Ever								
0–1	1.11	0.97	1.26	0.124				
2 or 3	ref	ref	ref	ref				
4+	1.11	0.96	1.28	0.165				

In this model, HIV status was not a significant predictor of unintended pregnancy (RR 1.14, CI: 0.96 to 1.35, *p* = .145). If the unintended pregnancy was after 2004 there was a lower risk for unintended pregnancy (RR = 0.75 CI: 0.66 to 0.84, *p* < .001). Women with a secondary education or higher also had a decreased risk in unintended pregnancy [RR = 0.70; CI: 0.70 to 0.92, *p* = 0.002]. Other traditional risk factors, such as age and parity, were also significant predictors of pregnancy intentions in the adjusted model.

### Multinomial logistic regression model:

Table 3 shows the multinomial logistic regression models comparing intended pregnancy with each type of unintended pregnancy. Due to the categorical outcome, the statistics presented in Table 3 are a relative risk ratio (RRR), which is interpreted in the same way as a risk ratio; therefore, a RRR above 1 indicates an increased risk for the outcome and a RRR below 1 indicates a decreased risk.

**Table 3:** Predictors of unintended pregnancy using multinomial logistic regression, Rakai community cohort study, Uganda 2001–2013

N = 2819												
RISK FACTORS	Mistimed v. Intended				Unwanted v. Intended				Ambivalent v. Intended			
	RRR	95% Lower	95% Upper	*P*-value*	RRR	95% Lower	95% Upper	*P*-value*	RRR	95% Lower	95% Upper	*P*-value*
Sample Characteristics												
Pre or Post ARV												
pre 2004	ref	ref	ref	ref	ref	ref	ref	ref	ref	ref	ref	ref
post 2004	0.68	0.55	0.83	<.001	0.31	0.23	0.41	<.001	0.51	0.29	0.88	0.016
Individual Characteristics												
Age												
15–19	1.91	1.33	2.74	<.001	1.27	0.70	2.28	0.430	1.20	0.45	3.23	0.720
20–24	1.20	0.96	1.50	0.113	0.36	0.23	0.57	<.001	0.58	0.29	1.17	0.129
25–29	ref	ref	ref	ref	ref	ref	ref	ref	ref	ref	ref	ref
30–34	0.87	0.67	1.13	0.294	2.13	1.52	2.98	<.001	0.52	0.23	1.22	0.133
35–39	0.88	0.60	1.30	0.517	3.50	2.31	5.29	<.001	1.04	0.43	2.55	0.927
40–49	0.80	0.34	1.89	0.607	9.36	4.47	19.59	<.001	7.97	2.72	23.34	<.001
SES												
low	ref	ref	ref	ref	ref	ref	ref	ref	ref	ref	ref	ref
middle	0.97	0.76	1.24	0.813	0.98	0.69	1.39	0.896	1.19	0.64	2.23	0.578
high	0.85	0.67	1.08	0.182	0.78	0.55	1.10	0.159	0.91	0.50	1.67	0.768
Education												
No school	1.24	0.78	1.96	0.370	1.48	0.81	2.73	0.203	1.54	0.53	4.48	0.430
Primary school	ref	ref	ref	ref	ref	ref	ref	ref	ref	ref	ref	ref
Secondary schol or more	0.68	0.55	0.84	<.001	0.66	0.47	0.93	0.017	0.82	0.45	1.48	0.506
HIV Status												
Positive	0.84	0.60	1.19	0.328	2.44	1.65	3.61	<.001	2.07	1.03	4.18	0.041
Parity												
0	0.39	0.27	0.58	<.001	0.37	0.16	0.84	0.018	0.65	0.21	2.02	0.456
1 or 2	ref	ref	ref	ref	ref	ref	ref	ref	ref	ref	ref	ref
3 or 4	1.48	1.15	1.90	0.002	2.20	1.43	3.38	<.001	1.08	0.50	2.31	0.844
5+	1.36	1.07	1.72	0.011	3.34	2.25	4.95	<.001	1.14	0.54	2.41	0.722
Marriage Status												
never married	1.47	1.06	2.04	0.022	1.41	0.80	2.49	0.234	0.93	0.34	2.57	0.893
married	ref	ref	ref	ref	ref	ref	ref	ref	ref	ref	ref	ref
separated or widowed	0.96	0.64	1.42	0.822	1.65	1.06	2.58	0.028	1.75	0.79	3.88	0.169
Number Sexual Partners Ever												
0–1	1.18	0.96	1.45	0.119	1.33	0.94	1.90	0.110	0.98	0.53	1.80	0.941
2 or 3	ref	ref	ref	ref	ref	ref	ref	ref	ref	ref	ref	ref
4+	0.86	0.66	1.12	0.273	1.24	0.88	1.74	0.229	0.79	0.39	1.59	0.502

HIV-positive women in the sample had a significantly increased risk for an unwanted pregnancy [RRR = 2.44, CI: 1.65 to 3.61, *p* < .001] and an ambivalent pregnancy [RRR = 2.07; CI: 1.03 to 4.18, *p* = 0.041]. HIV-positive women do not have a significantly different percentage of mistimed pregnancies. The interaction between the survey round taking place before or after 2004 and HIV status was not significant. Women with a secondary education or higher also had a reduced risk for both mistimed pregnancy [RRR = 0.68; CI: 0.55 to 0.84, *p* < .001] and unwanted pregnancy [RRR = 0.66; CI: 0.47 to 0.93, *p* < .001].

## Discussion

This paper demonstrates that woman’s HIV status remains an important predictor of unwanted pregnancy in Rakai, Uganda. Secondly, this paper underscores the importance of understanding the nuances of unintended pregnancy types for unwanted, mistimed, and ambivalent pregnancies. Finally, we found that pregnancy intentions in Rakai, Uganda have significantly changed over time, with unwanted and mistimed pregnancies having decreased over time. Perhaps this change is a result of ART availability which would make pregnancy and HIV infection safer or changing social conditions such as rising levels of education and socioeconomic status.

Previous literature is mixed on the impact of HIV status on pregnancy. Some studies show that HIV status does not impact the desire for children, while others show that knowing you are HIV positive reduces the desire for children [20–23]. This study extends previous research by demonstrating the importance of the types of unintended pregnancy when examining HIV as a risk factor. Not only does this study add to earlier findings regarding fertility desire and HIV-positive women but, it also extends these findings beyond fertility desire showing the effect on actual pregnancy.

When looking at unintended pregnancy dichotomously – intended vs. unintended – we see no significant effect. When examining the different types of unintended pregnancy, we see that HIV status has no significant effect on mistimed or ambivalent categories; however, after controlling for other predictors, HIV status has a significant effect on risk for unwanted pregnancy. Even with the increase in availability of treatment and the reduction of risk in terms of an HIV-woman giving birth to an HIV-positive baby, HIV status remains a predictor for unwanted pregnancy.

Between 2001 and 2013 there was a significant increase in prevalence of intended pregnancies in the study population. A number of factors may be contributing to this finding. During this time period, HIV treatments and prevention interventions have become more available such as prevention of mother to child transmission (PMTCT) in the early 2000s, antiretroviral treatment (ART) starting in 2004, and male circumcision (MC) in 2008. Increasing availability of HAART has reduced mortality in Uganda [24], and in 2014 it was estimated that over 95% of pregnant women living with HIV were receiving ART for prevention of mother-to-child transmission [25].

In context of the HIV epidemic in Rakai, these shifts in HIV prevention and treatment have made childbearing safer for HIV-positive women compared to the 1990s when HIV care and prevention options were limited. During this period, there was also increased fertility desire in the Rakai District. Between the years 2001 and 2011, women’s desire for children increased from 46% to 52% [14]. This change may also be the result of improved HIV programming and may partially explain why intended pregnancy was also increasing. In 2004, after ART became available in the region, our data demonstrates that there is decreased risk for unwanted pregnancies.

Other potential factors in the rise of intended pregnancy and decline in unintended pregnancy are social factors such as increasing wealth and rising levels in school enrollment. In Uganda, the national policy of Universal Primary Education (UPE) was started in 1997, which eliminated tuition fees and increased access for young women [26]. In our sample, the number of women with a secondary education or higher was rising as was socioeconomic status which is measured by household assets. These changing trends may also explain part of the increase in intended pregnancy [27]. Increased access to education influences reproductive health in many ways: delaying workforce entrance, family formation, and potentially delaying initiation of sexual intercourse and childbearing [28]. Increasing school enrollment and rising SES are associated with lower risk for HIV and pregnancy [29]. Since a large percentage of unintended pregnancies are among young women, and young women are now staying in school longer, this may be one of the driving forces behind the shifting demographics in intended pregnancy over time in Rakai, Uganda.

### Limitations

We would note several limitations in our study. We were not able to identify which type of contraceptive use was used at the time of the pregnancy or the outcomes for these pregnancies. Knowing which contraceptive methods were being used, if any, and how they correlate with different types of unintended pregnancy may allow for more in-depth policy solutions regarding family planning methods.

We also do not know if the individual women are on ART before or during their pregnancies. Again, knowing ART status would allow us to better understand if the influence of ART is having an effect on unintended pregnancy at the individual level. Or, if it is reducing unintended pregnancy on a community level due to less stigmatization of HIV and better sense of treatment and outcomes.

Our measurement of unintended pregnancy did contain multiple categories, but did not measure even more specific aspects of mistimed pregnancies such as by how much time a pregnancy was unintended. Further research should examine if there are differences among women who are mistiming pregnancies by large time periods versus shorter time periods.

We were unable to consider abortion or attempted abortion in our study. Due to the illegality of abortion in Uganda, and the sensitivity of the topic, abortion was only asked about in one survey round and had considerable missing data. It will be important in future studies to understand how women who attempt or complete abortions affect these findings. Finally, we know that men and parents influence pregnancy intentions; however, the men in our sample were not asked about intendedness of previous children. Therefore, we have written a follow-up paper using qualitative data that looks at interviews done with dyads that explore pregnancy intendedness from both the male and female perspective.

## Conclusion

We found important changes in unintended pregnancy overtime coinciding with a changing context of ART availability and changes in social context. Ongoing efforts are needed to prevent both HIV infection and unintended pregnancy. HIV was associated with unwanted pregnancy and access to ART. Education may be augmenting prevention of unintended pregnancy. Access and education regarding family planning to HIV-positive women regarding long acting or permanent methods of contraception may be an appropriate policy implication using these findings. Improving social conditions such as increasing educational opportunities for women and reducing poverty is another broader and long term intervention. Finally, in order for interventions to effectively target women for improved family planning, it is may be important to understand the nuances of each type of unintended pregnancy. For example women reporting mistimed pregnancies may need reversible contraception where as women reporting unwanted pregnancies who do not want more children may need to receive counseling on more permanent birth control methods.

## Abbreviations

ART: Antiretroviral therapy
CI: Confidence interval
GEE: Generalized estimating eqs.
HIV: Human immunodeficiency virus
IRB: Institutional Review Board
MC: Male circumcision
PMTCT: Prevention of mother-to-child transmission (PMTCT)
RCCS: Rakai Community Cohort Study
RRR: Relative Risk Ratio
SES: Socioeconomic status
STI: Sexually transmitted infection
